# Using Machine Learning Multiclass Classification Technique to Detect IoT Attacks in Real Time

**DOI:** 10.3390/s24144516

**Published:** 2024-07-12

**Authors:** Ahmed Alrefaei, Mohammad Ilyas

**Affiliations:** Electrical Engineering & Computer Science, Florida Atlantic University, 777 Glades Road, Boca Raton, FL 33431, USA; aalrefaei2021@fau.edu

**Keywords:** Internet of Things, intrusion detection system, machine learning, PySpark architecture

## Abstract

This paper presents a real-time intrusion detection system (IDS) aimed at detecting the Internet of Things (IoT) attacks using multiclass classification models within the PySpark architecture. The research objective is to enhance detection accuracy while reducing the prediction time. Various machine learning algorithms are employed using the OneVsRest (OVR) technique. The proposed method utilizes the IoT-23 dataset, which consists of network traffic from smart home IoT devices, for model development. Data preprocessing techniques, such as data cleaning, transformation, scaling, and the synthetic minority oversampling technique (SMOTE), are applied to prepare the dataset. Additionally, feature selection methods are employed to identify the most relevant features for classification. The performance of the classifiers is evaluated using metrics such as accuracy, precision, recall, and F1 score. The results indicate that among the evaluated algorithms, extreme gradient boosting achieves a high accuracy of 98.89%, while random forest demonstrates the most efficient training and prediction times, with a prediction time of only 0.0311 s. The proposed method demonstrates high accuracy in real-time intrusion detection of IoT attacks, outperforming existing approaches.

## 1. Introduction

The Internet of Things (IoT) comprises a network of interconnected objects that gather and exchange data through the Internet with other devices and systems. These objects range from everyday items, such as smartwatches and household appliances to vehicles and industrial machinery [1]. IoT aims to enhance the productivity and connectivity by automating and enhancing numerous processes and tasks. Additionally, IoT is seen as a technology and innovation poised to revolutionize industries such as smart homes, advanced healthcare, efficient transportation, and precision agriculture. Within IoT, diverse physical objects can collaborate and communicate autonomously to transmit data across various networks without requiring human intervention. IoT devices have outpaced non-IoT devices on a global scale. In 2022, there were 21.7 billion active connections, representing 54% of the total IoT devices [2]. Also, a new study by Cisco estimates that 500 billion devices will have access to the Internet by 2030 [3]. This exponential expansion of interconnected devices provides both enormous opportunities and significant challenges. Different vulnerabilities present in IoT devices, such as weak passwords and inadequate encryption attackers often exploit these weaknesses to launch various cyber-attacks [4]. As more devices become interconnected, and widespread use in various areas of our lives has made them attractive targets for attackers. Hackers are constantly exploring new ways to launch advanced attacks. In 2025, more than 25% of all attacks will target IoT devices [5]. As attackers increasingly focus on IoT devices, it is essential to have efficient intrusion detection mechanisms. The implementation of intrusion detection systems (IDSs) in IoT has grown imperative due to the increasing number of interconnected devices. The main focus of IDS is to examine network traffic and detect cyber-attacks. Through the analysis of data collected from different IoT devices, an IDS can identify the anomalous activity or illegal entry, therefore securing the network and its connected devices against cyber-attacks [6]. A significant obstacle in employing IDS in IoT is the heterogeneous characteristics of devices in IoT and the substantial amount of data that has been produced. Conventional IDS may have difficulties in managing the large size of data and the diverse range of communication protocols employed in the IoT. Advancements in machine learning have facilitated the development of more advanced IDS that can adjust to the ever-changing IoT. Machine learning (ML) techniques have been introduced as promising methods for real-time intrusion detection to detect various types of attacks in IoT environments, offering the advantage of adaptability to evolving attack strategies and patterns. The use of real-time in IDS refers to continuously monitoring and analyzing IoT network traffic and device behavior to detect unusual or malicious activities in real time. It is essential in IoT for timely response and adaptability to dynamic environments. Real-time intrusion detection allows for a fast response to security attacks, reducing the potential damage affected by attacks [7]. It has an advantage over classical IDS systems which may experience latency due to the batch processing of data having limited adaptability, relying on predefined rules, and employing static detection mechanisms, resulting in delayed responses and reduced effectiveness in detecting emerging threats. Real-time IDS systems are being developed to address these limitations to provide faster and more adaptive detection capabilities for IoT. Different approaches use real-time for effective attack detection. For instance, anomaly detection establishes normal behavior baselines by analyzing the historical data or observing typical patterns in network traffic and device behavior [8]. This method continuously monitors activity, flagging any deviations as potential intrusions or threats, though it requires accurate modeling to minimize false positives. Another approach that uses real-time is signature-based detection which swiftly identifies known attack patterns by comparing arriving traffic against signatures in a database [9], offering quick and accurate detection but potentially struggling with novel or zero-day attacks [10]. Also, behavioral analysis techniques can be used to closely scrutinize real-time behaviors, detecting anomalies that may indicate security breaches, with the advantage of adaptability to evolving attack strategies but requiring sophisticated algorithms and the accurate profiling of normal behavior. This paper presents a novel approach to real-time intrusion detection using the Hadoop Spark framework in the context of IoT. We used the IoT-23 dataset which is a dataset that contains IoT network traffic from different types of IoT devices. Our IDS system employs PySpark, an interface for Apache Spark in Python, for efficient data processing and analysis. Central to our methodology is the utilization of the One-vs-Rest (OVR) multiclass classification technique, enabling the accurate categorization of network traffic and the detection of various attack types in real-time. The OVR approach decomposes the multiclass problem into several binary classification tasks, allowing for tailored optimization for each attack type and enhancing overall detection accuracy. This method simplifies the classification process, making it easier to manage and interpret. Additionally, we integrate spark streaming to facilitate real-time data processing, ensuring that the IDS can handle continuous streams of network data and provide the timely detection of threats. Furthermore, our approach incorporates synthetic minority oversampling (SMOTE) to address class imbalance and SelectKBest for feature selection, enhancing the accuracy and reliability of our models. We implement and evaluate multiple machine learning algorithms supported by PySpark—decision trees (DT), random forest (RF), logistic regression (LR), and extreme gradient boosting (XGB)—comparing their performance in terms of accuracy and response time. To assess the effectiveness of our IDS, we employ standard evaluation metrics including precision, recall, and F1 score. Our results demonstrate a detection accuracy of up to 98.89% and minimal prediction latency, validating the efficacy of our real-time approach in IoT environments.

Our contribution can be summarized as follows:

- We present an effective IDS designed specifically for the IoT environment that demonstrates high accuracy in detecting and classifying various types of attacks. The scheme utilizes PySpark’s architecture and machine learning algorithms for processing real-time data. By integrating these powerful tools, our IDS achieves superior accuracy and efficiency in real-time threat detection and classification.
- We utilize synthetic minority oversampling strategies (SMOTE), an oversampling method, to address the class imbalance in our dataset. Additionally, we employ feature selection techniques such as SelectKBest to identify most relevant features for our model.
- We implement and test five machine learning models that PySpark supports and provide a comparison between different machine learning IDS and our approach. The main comparison includes the accuracy of each model and the response time.
- To evaluate our approach, we used the evaluation metrics such as accuracy, recall, precision and F1 score. Our highest detection accuracy is 98.89% and lowest prediction with 0.0311 s.

The following sections of this study are organized as follows: Section 2 discusses the literature review. Section 3 provides an overview of Apache Spark, machine learning methods, OVR, and PySpark architecture. In Section 4, we focus on our proposed scheme. Section 5 is the result and analysis. Section 6 includes the conclusion and future works.

## 2. Related Work

This section provides a relevant study on IDS in IoT network. In [11], the authors suggest employing real-time machine learning and deep learning techniques to identify anomalies within IoT networks. Algorithms such as DT were used in their research paper to offer the highest accuracy in detecting these anomalies, as demonstrated through testing on the IoT-23 dataset. Their study assessed both the performance and time requirements of various models to determine the most effective algorithm for anomaly detection. They achieved 73% detection accuracy and 3 s prediction time. Another study [12] proposes the use of the support vector machine (SVM)-based detection of distributed denial of service (DDoS) incidents in IoT, utilizing the same dataset. DDoS attacks present a significant threat to smart cities networks by overwhelming targeted servers, services, or networks with a flood of network traffic. In their research, they recommend using ML algorithms, such as SVM, DT, and RF, which are trained and evaluated for the classification of DDoS-affected network packets. Principal component analysis (PCA) is employed in their paper to enhance the performance of these algorithms. Their experimental analysis demonstrates that incorporating the PCA notably reduces the algorithm’s execution time while maintaining comparable results with reduced features. The DT and RF algorithms exhibit superior accuracy in classifying DDoS packets compared to SVM. Furthermore, Bhandari, Lyth, Shalaginov, and Grønli [13] introduce an innovative framework designed to identify malware attacks within smart IoT ecosystems using artificial intelligence (AI) techniques. Their framework’s architecture caters to diverse situations, effectively surveilling network activity to detect malware- and IoT-based attacks. It conducts thorough performance and concurrency assessments on a deep neural network (DNN) model implemented across IoT devices. The deployment of this DNN model on two IoT gateways showed encouraging results, with minimal impact on network bandwidth (less than 30 kb/s on average) and only a 2% rise in CPU usage. The machine learning models exhibited a high level of accuracy, achieving nearly 93% detection accuracy and 0.2 s prediction time across different datasets. Their findings highlight the framework’s effectiveness in accurately and efficiently detecting malware and attacks in smart environments. This paper [14] presents the edge-cloud deep IDS model, a new method for IoT within the Lambda architecture. Traditional intrusion detection systems (IDSs) often struggle with scalability, storage, processing, and security concerns when faced with enormous volumes of data generated by the Internet of Things (IoT). This innovative method aims to directly tackle these issues. By incorporating deep learning techniques, the proposed model enhances both the training phase duration and the accuracy in identifying true positive attacks. When compared to more traditional machine learning approaches, the performance and adaptability offered by neural network layers are greater in term of performance. The suggested system not only lets one spot suspicious activity in real time, but it also lets you examine historical data in a batch process to categorize it. A distributed and real-time DDoS detection system called S-DDoS was proposed by [15] as a way to identify DDoS attacks. It is built on the Apache Hadoop framework and uses streaming technology. By utilizing the K-Means clustering algorithm, their system was able to detect DDoS attack traffic with a high degree of accuracy in real-time. Another work [16] looked on efficient Spark-based anomaly detection using the UNSW-NB15 dataset. Using Spark for anomaly identification, the authors demonstrated that their suggested strategy yielded better accuracy. The authors in [4] employ ensemble learning techniques, particularly AdaBoost, and integrate various feature selection methods. Their model has been tested on different datasets including the IoT-23 using GPU, showing a high performance with an accuracy of nearly 99.9% and 0.02156 s for detection time.

## 3. Background

In this section, we introduce key components essential for understanding the context of our study. We delve into the concepts of intrusion detection systems (IDSs), multiclass classification, and Apache spark, explaining their significance and relevance to the study.

### 3.1. Intrusion Detection System (IDSs)

One of the critical security approaches used is an intrusion detection system (IDS). The concept of IDS was proposed in1980 [17]. Traditional intrusion detection systems (IDSs) are insignificant in the IoT environment due to their heterogeneity, abnormally high levels of normal behavior, and the exponential increase in vulnerabilities brought on by the growing number of Internet of Things (IoT) [18]. Intrusion detection systems can be categorized into two main types: network-based IDS (NIDSs) and host-based IDSs (HIDSs). NIDSs monitor network traffic, examining the packets traversing the network for suspicious patterns, while HIDSs focus on individual devices, examining their activities and configurations for signs of compromise [19]. Both types of IDS are seen using real time as an innovative approach that enhances IDS strategy.

### 3.2. Machine Learning Multiclass Classification

An essential part of machine learning is multiclass classification, the process of sorting data into specific categories. When more than two classes are present, multiclass classification takes over from binary classification, which only works with two classes. Effectively describing the relationships between input features and many output classes is the challenge of multiclass classification. DT, RF, XGB, LR, KNN, and NB are just a few of the techniques that can be employed for this specific task. It is usual practice to expand the binary classification algorithms to address multiclass situations using approaches such as one-vs.-all (OvA) and one-vs.-one (OvO). Among the many fields that see multiclass classification in action are cybersecurity, image recognition, and natural language processing. In the field of cybersecurity, multiclass classification can be useful for dividing network traffic into distinct threat types, which in turn can help with the early detection and prevention of cyber-attacks.

### 3.3. Apache Spark

Apache spark is a distinguished as big data framework that excels at advanced analytics, real-time streaming, graph processing, machine learning, and batch processing. There are noticeable performance benefits compared to Hadoop due to its in-memory data processing capabilities [20]. Spark’s popularity has risen alongside the growth of the use of real-time IDS applications. While primarily Scala-based, it provides APIs for Java, Python, and R, enhancing its accessibility. Data can be cached in memory with Spark’s introduction of resilient distributed datasets (RDDs), which significantly cuts down on the frequency of disk reads [20]. By converting incoming data into discretized streams (DStreams), spark streaming expands its capabilities even further. This allows for fast and fault-tolerant real-time stream processing [21].

#### PySpark

**Figure 1:**
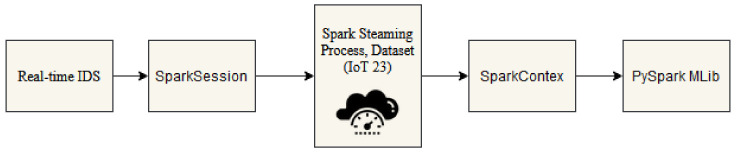
PySpark architecture.

A variety of algorithms designed for various applications, such as machine learning, are part of PySpark [22], which is a component of Apache Spark’s environment. A few algorithms are available in PySpark’s MLlib. These include decision tree (DT), extreme gradient boosting (XGBoost), random forest (RF), gradient boosted tree (GBT), and linear support vector machine (SVM), among others. The range of methods available for distributed data processing and analysis within the Spark framework is expanded by PySpark’s continuous integration with other Python libraries such as NumPy, pandas, and scikit-learn. In this architecture, the primary goal is to swiftly detect anomalies or potential security breaches in real-time data streams, particularly focusing on IoT data. The system begins with the initiation of a SparkSession, serving as the entry point to the PySpark application. Spark Streaming processes the continuous data streams, coordinating their execution through SparkContext, which manages distributed data processing and resource allocation across the cluster. Subsequently, PySpark MLlib is employed for real-time anomaly detection, utilizing various machine learning algorithms such as DT, KKN, LR, RF, and XGB. This architecture integrates Spark’s streaming capabilities with MLlib’s machine learning tools, facilitating scalable and efficient intrusion detection in IoT data streams. The PySpark architecture is represented in Figure 1.

## 4. Proposed Method

**Figure 2:**
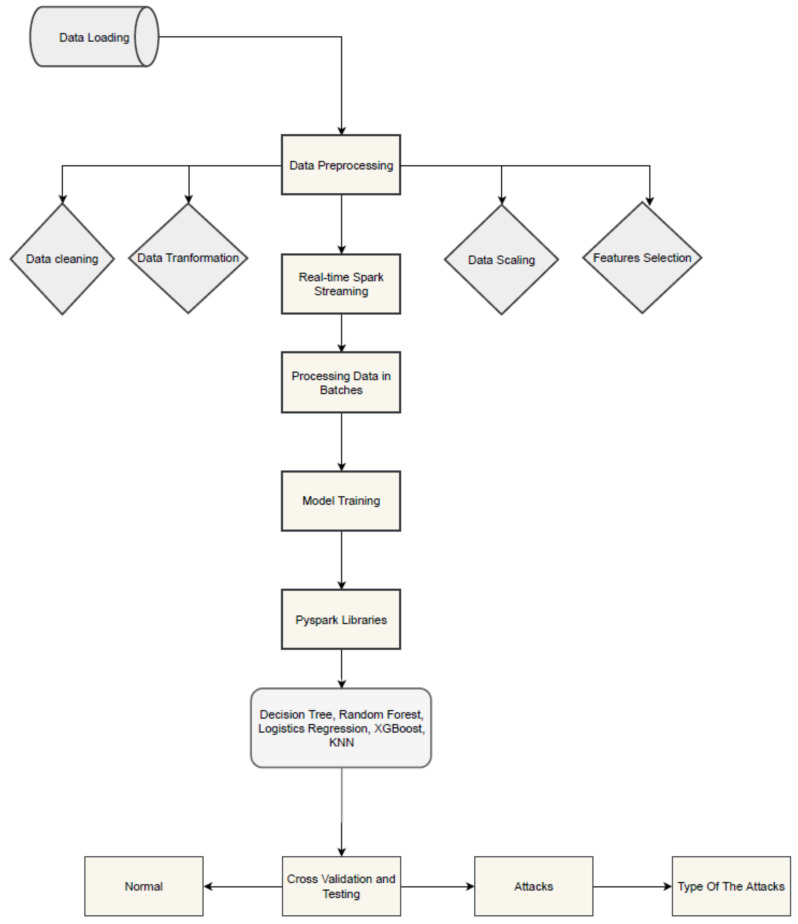
Preparation framework for real-time IoT intrusion detection systems using PySpark architecture.

This part of the paper proposes a real-time IDS for detecting IoT attacks using multiclass classification models. Five ML algorithms have been used in this paper: multiclass decision tree (DT); random forest (RF); extreme gradient boosting (XGB); logistic regression (LR); and K-nearest neighbors (KNN) using the OneVsRest (OVR) technique. The proposed approach focuses on the use of real-time IDS in IoT using PySpark architecture. The scheme’s main purpose is to enhance the detection accuracy in real-time and reduce the prediction time. The OVR technique is used to classify the IoT network traffic into normal and attack categories, including DDOS attack, Okiru attack, horizontal port scan fragment attack, and other benign traffic present in the IoT-23 dataset. Several critical procedures are implemented when preparing the dataset for use in real-time intrusion detection systems (IDSs) with the PySpark libraries, as presented in Figure 2. To train the IoT-23 dataset, particularly for attack data, we follow these steps: data acquisition, obtaining the IoT-23 dataset, which contains a variety of network traffic data labeled as normal or various attack types. Data preprocessing, where data scaling is used to normalize the feature values to ensure uniformity in scale, which helps improve the performance of certain algorithms. Feature selection is performed to identify and select the most relevant features that contribute to distinguishing between normal and attack traffic. The synthetic minority oversampling technique (SMOTE) is applied to generate synthetic samples for underrepresented attack classes, addressing class imbalance and improving the model performance. Data Transformation tools are used to encode categorical variables into numerical formats using techniques like one-hot encoding and transform-skewed distributions to approximate normality. Data cleaning techniques are employed to remove or input missing values, handle outliers, and filter noise to ensure high-quality data. In the model training phase, the OneVsRest classification is used to train each of the five ML algorithms (DT, RF, XGB, LR, KNN) to handle the multiclass classification problem. Each model is trained to differentiate one class (either normal or one of the attack types) against all other classes. Model validation uses cross-validation techniques to tune hyperparameters and evaluate model performance to prevent overfitting and ensure generalizability. Finally, in model evaluation, the models are assessed using metrics such as precision, recall, F1 score, and accuracy to evaluate their effectiveness in detecting various attack types and normal traffic. The performance of the different models is compared to determine the most effective approach for real-time intrusion detection in IoT environments. By following these steps, we ensure that the IoT-23 dataset is properly processed and utilized for training robust models capable of detecting a wide range of IoT attacks in real-time [23,24,25,26,27]. This ensures that the dataset is of high quality and improves its effectiveness in real-time intrusion detection activities.

### 4.1. Data Collection

The original data utilized in this research are from the IoT-23 dataset, published in January 2020 [28]. This dataset comprises network traffic gathered from three distinct smart home IoT devices: Philips HUE, Somfy Door Lock, and Amazon Echo. It is a comprehensive dataset containing both real and labeled instances of IoT attacks, as well as benign traffic, specifically designed for the development of machine learning algorithms. The dataset comprises 23 captures, with 20 instances of malicious captures and three instances of benign captures. Each capture from infected devices may include the name of the executed malware sample. The malware classifications within the IoT-23 dataset include Attack, Command and Control (C & C), C & C File Download, C & C Heartbeat, C & C Heartbeat Attack, C & C Heartbeat with File Download, C & C Mirai, C & C Torii, Distributed Denial of Service (DDoS), File Download, Okiru, Okiru Attack, and Horizontal Port Scan Fragment. The number of attacks and normal traffic is shown in Table 1. There is also a small version of this dataset named IoT 23 Combined, which is provided in this paper [11], and it contains 1,444,674 records. Our research focuses on this shorter version of the IoT 23 with four different traffic in IoT 23 dataset which are the DDOS attack, Okiru attack, Horizontal Port Scan Fragment attack, Attack and Benign traffic. We named the Attack label as unknown attack in the IoT 23 traffic.

### 4.2. Data Preprocessing

Data preprocessing involves four important steps: data cleaning, data transformation, data scaling, and SMOTE.

#### 4.2.1. Data Cleaning

Data cleaning is the process of identifying and correcting errors, inconsistencies, and inaccuracies in a dataset to ensure its integrity and reliability for analysis. We use several techniques aimed at detecting and addressing various types of issues, such as missing values, outliers, duplicate entries, and inconsistencies in formatting or labeling.

#### 4.2.2. Data Transmission and Data Scaling

Data transformation involves altering the structure or layout of data to enhance its suitability for analysis, modeling, or visualization purposes. In our research paper, we employed techniques like normalization and categorical variable encoding. Normalization is important in preparing datasets for machine learning algorithms by standardizing numeric features, such as sensor readings or device parameters, to a consistent range, typically between 0 and 1 or −1 and 1. This ensures that no single feature unduly influences the analysis due to its scale, especially when dealing with IoT sensor data from diverse devices with varying measurement scales. The other technique that we used is encoding, which converts categorical variables into numerical representations, such as binary indicators through one-hot encoding. This enables IoT analysts to integrate these variables into machine learning models effectively.

#### 4.2.3. Synthetic Minority Over-Sampling Technique (SMOTE)

We employed data balancing techniques during the model training process to address the issue of data imbalance between different classes in our dataset. Specifically, we used SMOTE, a method utilized in machine learning to tackle class imbalance by generating synthetic samples from the minority class. SMOTE works by randomly selecting minority class instances and creating new instances along the line segments between these instances and their nearest neighbors. By interpolating new instances, SMOTE effectively increases the representation of the minority class without duplicating existing data points, thus mitigating the impact of class imbalance on model performance [29]. However, the effectiveness of SMOTE can vary depending on factors such as dataset characteristics and the choice of parameters, and careful consideration is necessary to avoid introducing the noise into the dataset.

#### 4.2.4. Features Selection

This study involved feature selection techniques to improve our machine learning models’ predictive accuracy and interpretability. We utilized three distinct methods: SelectKBest, Select From Model with XGB, and Select From Model with Random Forest. The ease and efficiency of SelectKBest in identifying the most relevant characteristics using statistical tests led to its selection. To find the features that are most strongly associated with the target variable, SelectKBest evaluates each separately. This might help decrease overfitting and improve the accuracy of the model. In addition, “Select From Model” was implemented with XGB and random forest, which were recognized for their robustness in handling complex data. By using the feature importances provided by these ensemble methods, Select From Model automatically selects the most relevant features for prediction. This approach capitalizes on the inherent ability of ensemble methods to capture intricate relationships within the data, thereby improving the predictive performance. These methods were chosen since they combine effectively and address distinct aspects of feature selection. For preliminary screening, we used Select From Model with XGB and RF, which makes use of sophisticated machine-learning models. By combining these approaches, we aim to create a feature subset that maximizes prediction accuracy, reduces prediction time, and enhances model interpretability and generalization.

### 4.3. Machine Learning Algorithms

In the real-time detection of IoT attacks, a PySpark architecture has been utilized to employ multiple machine learning models: decision trees (DTs), random forests (RF), extreme gradient boosting (XGB), logistic regression (LR), and K-nearest neighbors (KNN). Each of these algorithms employed a multiclass classification technique to analyze the data and identify potential security attacks in real-time within the PySpark architecture.

#### 4.3.1. Decision Tree (DT)

DT serves as a useful tool in machine learning, particularly in regression and classification tasks. With the aim of training models to predict attacks based on limited data points, decision tree algorithms employ a tree-like structure consisting of decision nodes and leaves. These nodes facilitate the partitioning of data into smaller branches, while the leaves represent the final outcomes, determining whether specific dataset traffic constitutes an attack. This method proves effective for both classification and regression problems [13]. Entropy, a measure of uncertainty in data samples, plays a crucial role in decision tree construction. Furthermore, decision trees implemented in PySpark can be utilized in real-time IDS, deploying the distributed computing capabilities of PySpark to swiftly analyze and classify IoT network traffic data as attacks occur. Additionally, PySpark’s decision tree implementation supports One-Versus-Rest (OVR) multiclass classification, enabling the classifier to handle scenarios with multiple classes by training multiple binary classifiers, each distinguishing between one class and the rest. This capability enhances the versatility of decision trees, making them suitable for the complex classification tasks encountered in real-time IDS applications.

#### 4.3.2. Random Forest (RF)

RF was introduced by this ensemble algorithm in [17], which combines decision trees and bagging techniques (DT) by constructing a “forest” of decision trees. In each tree node, a property is randomly chosen to serve as a decision boundary. This approach addresses overfitting issues commonly encountered with decision trees. The bootstrap procedure [17] is utilized to generate sample sizes from the original data source. The RF classifier, a variant of the decision tree method, extends the Random Forest algorithm to handle multiple classes in classification tasks. In this variant, decision trees are trained with subsets of input features and training data, and the input data’s class is predicted by aggregating the classes predicted by individual trees. The Spark.ML implementation offers RF for both binary and multiclass classification, as well as for regression tasks, accommodating both continuous and categorical features [22].

#### 4.3.3. Logistic Regression (LR)

LR serves as both a linear regression and a classification algorithm, particularly adept at predicting binary outcomes. It operates by generating a logistic curve, restricting its output values to fall between 0 and 1. LR’s utility extends further through its adaptation for multiclass classification using the OVR approach [30]. In situations where a single LR model’s efficacy is limited or the dataset complexity necessitates multiple models to capture diverse data features, this approach becomes particularly valuable. In Spark.ML, logistic regression is employed for binary outcome prediction via binomial logistic regression or for multiclass outcome prediction through multinomial logistic regression [31].

#### 4.3.4. Extreme Gradient Boosting (XGB)

XGB, a highly successful tree-boosting technique, represents one of the newer ensemble machine-learning algorithms that has demonstrated remarkable performance across various domains [11]. Operating as a sequential ensemble method, XGB constructs models similar to decision trees. Each data value in the dataset is assigned a weight, dictating its influence on the decision tree’s selection process. Initially, all data values carry equal weight, which is then adjusted based on the analysis outcomes. The iterative process begins with the first round of data values, informing the creation of a new classifying model that builds upon the insights gained from the previous round; this process iterates until a reliable classifier is attained [29]. XGB is capable of handling multiclass classification tasks using the softmax objective. Within XGB, the softmax objective performs effectively in scenarios where each instance is assigned to only one class. The objective function aims to minimize the negative log-likelihood of the expected probability for each class. Spark ML does not fully support XGB, therefore we downloaded the jar files [32] and used them through the Juypter notebook.

#### 4.3.5. K-Nearest Neighbors (KNN)

K-Nearest Neighbors (KNN) algorithm functions differently from machine learning techniques such as DT or RF. It does not build a model as such; rather, it retains all the existing data and when a new instance comes in, KNN assigns it to a category that is most common among its nearest counterparts based on a certain measure of closeness, commonly the Euclidean distance [33]. Upon the advent of new information, KNN locates the closest neighbors using a predefined distance metric and infers the category for the new instance from these neighbors, in the case of classification tasks. KNN augments the capabilities provided by other models such as Decision Trees, Random Forests, Logistic Regression, and Extreme Gradient Boosting by providing an alternative approach to classifying IoT network traffic for security analysis. It has the potential to uncover insights that may be overlooked by other models, thus offering a broader view for detecting potential security incidents.

## 5. Result and Discussion

In this study, we utilized PySpark, a distributed computing framework, to implement an intrusion detection system (IDS) for real-time IoT botnet detection using the IoT-23 dataset. We employed various machine learning classifiers, including Logistic Regression (LR), Decision Tree (DT), Random Forest (RF), K-Nearest Neighbors (KNN), and Extreme Gradient Boosting (XGB). The performance of these classifiers was evaluated in terms of precision, recall, F1 score, and accuracy. These metrics provide insights into how well the model is performing. Our research specifically targeted DDoS (1) attacks, Okiru (2) attacks, Horizontal Port Scan Fragment (3) attacks, and unknown (4) attacks in the IoT-23 dataset and the differentiation from benign traffic (0). Accuracy measures the ratio of correctly predicted instances to the total number of instances in the dataset, providing an overall assessment of the model’s correctness. While precision focuses on the correctness of positive predictions, indicating the reliability of the model when it predicts a positive instance. It measures the proportion of true positive predictions among all positive predictions made by the model. Both Equations (1) and (2) are presented below:

(1) $$\mathrm{Accuracy}=\frac{\mathrm{TP}+\mathrm{TN}}{\mathrm{TP}+\mathrm{TN}+\mathrm{FP}+\mathrm{FN}}$$

where TP represents True Positive, TN represents True Negative, FP represents False Positive, and FN represents False Negative.

(2) $$\mathrm{Precision}=\frac{\mathrm{TP}}{\mathrm{TP}+\mathrm{FP}}$$

where TP represents true positive and FP represents false positive.

Recall emphasizes the model’s ability to capture all positive instances in the dataset. It measures the proportion of true positive predictions among all actual positive instances. On the other hand, the F1-Score is the harmonic mean of precision and recall, providing a balanced measure that considers both precision and recall. It is particularly useful when there is an uneven class distribution or when both false positives and false negatives are equally important. Recall and F1 score Equations (3) and (4) are presented below:

(3) $$\mathrm{Recall}=\frac{\mathrm{TP}}{\mathrm{TP}+\mathrm{FN}}$$

where TP represents true positive and FN represents false negative.

(4) $$F1=\frac{2\times (\mathrm{Precision}\times \mathrm{Recall})}{\mathrm{Precision}+\mathrm{Recall}}$$

These evaluation metrics collectively offer a comprehensive understanding of all models’ performance, enabling informed decision making in model selection and optimization. Additionally, we compared our results with existing works in the field to provide context and insights into the effectiveness of our approach.

### 5.1. Logistic Regression (LR)

**Figure 3:**
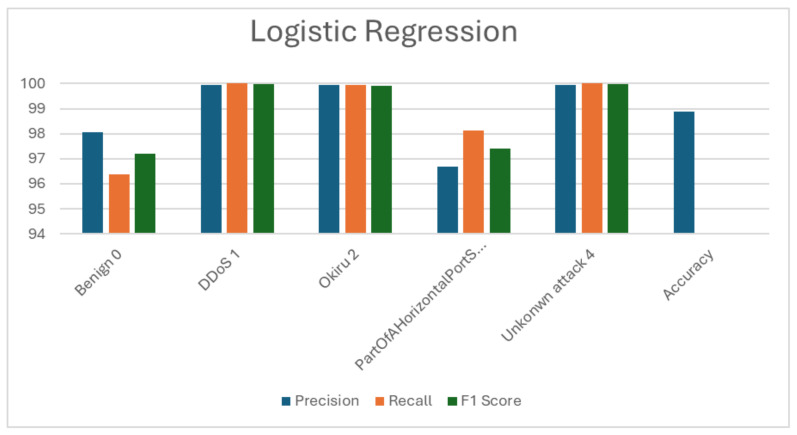
Visualization of logistic regression (LR) evaluation metrics.

The LR model achieved an overall accuracy of 82.98% on the dataset, with training taking approximately 95.6417 s and prediction only 0.4987 s. These results are summarized from Table 2 and Table 3, and Figure 3 provides a visual representation of the F1 score, precision, recall, and accuracy metrics. In terms of the class-specific performance, the model excelled in detecting DDoS and unknown attacks, achieving high precision and recall rates, resulting in high F1 scores for these classes. However, it struggled with accurately identifying benign instances and Part of A Horizontal Port Scan attacks, as indicated by the lower precision and recall rates for these classes. Specifically, the model exhibited high precision but low recall for benign instances, while it showed the opposite trend for part of a horizontal port scan attacks. Okiru attacks were detected with good precision and recall rates, resulting in a balanced F1 Score.

### 5.2. Random Forest (RF)

**Figure 4:**
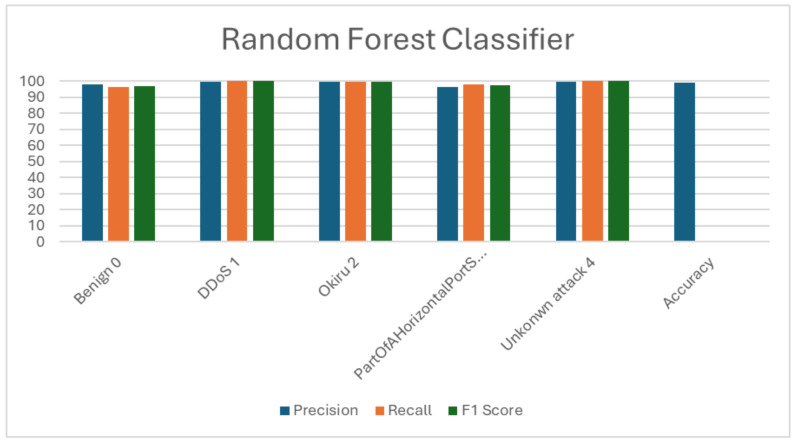
Visualization of random forest (RF) evaluation metrics.

RF delivered results with an overall accuracy of 98.54%. It required 30.86 s for training and a mere 0.0311 s for prediction. These findings are outlined in Table 4 and Table 5 while Figure 4 visualizes the F1 score, precision, recall, and accuracy metrics. Across all classes, the classifier showcased an exceptional performance, demonstrating high precision, recall, and F1 scores for each category. It particularly excelled in identifying DDoS and unknown attacks, achieving almost flawless precision, recall, and F1 scores. Moreover, the model accurately distinguished between the benign instances and part of a horizontal port scan attack, displaying strong precision, recall, and F1 scores for these categories. In summary, RF outperformed the LR model, underscoring its efficacy in accurately categorizing network traffic in IoT 23 dataset.

### 5.3. Decision Tree (DT)

**Figure 5:**
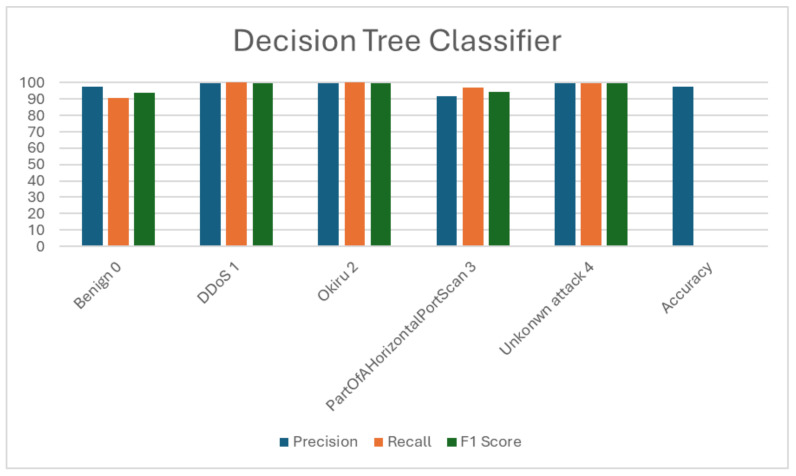
Visualization of decision tree (DT) evaluation metrics.

DT performance outlined in Table 6 and Table 7 indicates a strong capability in discriminating between different network traffic types, including benign and malicious activities, achieving an overall accuracy of 97.61%. The model reports high precision for benign traffic at 97.40% and nearly perfect precision and recall for DDoS (99.86%) and Okiru attacks (99.62%). Additionally, it effectively captures the majority of horizontal port scans. These metrics, alongside the F1 scores, precision, recall, and accuracy, are graphically detailed in Figure 5, reflecting the model’s suitability for efficient real-time intrusion detection.

### 5.4. K-Nearest Neighbors (KNN)

**Figure 6:**
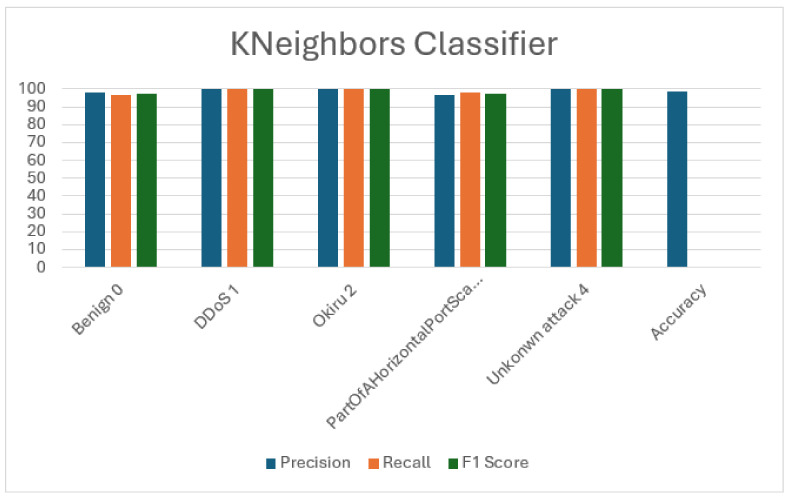
Visualization of K-Nearest Neighbors (KNN) evaluation metrics.

K-Nearest Neighbors (KNN) achieved a total accuracy of 98.87%. Training took 10.9469 s, with prediction requiring 0.9469 s. Across all classes, the classifier demonstrated high precision, recall, and F1 scores, excelling particularly in detecting DDoS and unknown attacks. While its accuracy rivals that of previous classifiers, its longer training and prediction times may affect its suitability for real-time applications compared to other classifiers. Nonetheless, KNN proves to be a strong performer in accurately categorizing network traffic. These results are detailed in Table 8 and Table 9, while Figure 6 visually represents the performance metrics.

### 5.5. Extreme Gradient Boosting (XGB)

**Figure 7:**
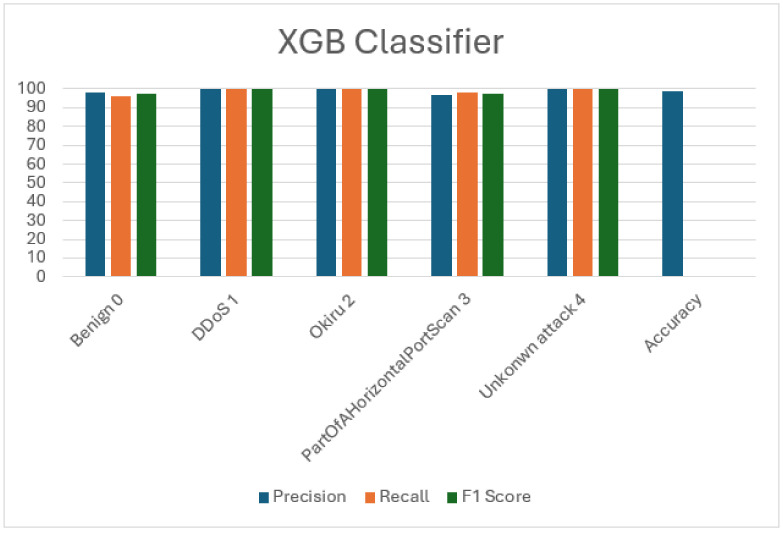
Visualization of Extreme Gradient Boosting (XGB) evaluation metrics.

XGB classifier demonstrates exceptional performance in classifying IoT network traffic data, boasting an impressive overall accuracy of 98.89%. Training the model required 50.3426 s, with prediction being executed in 0.7381 s. Detailed results are presented in the provided Table 10 and Table 11, while Figure 7 illustrates precision, recall, F1 score, and accuracy metrics visually. Across all classes, the classifier displayed a good precision, recall, and F1 scores, particularly excelling in detecting DDoS and unknown attacks. Notably, it achieved near-perfect precision, recall, and F1 scores for these categories. Additionally, the model accurately identified benign instances, Okiru attacks, and Part Of A Horizontal Port Scan attacks with robust precision, recall, and F1 scores.

**Figure 8:**
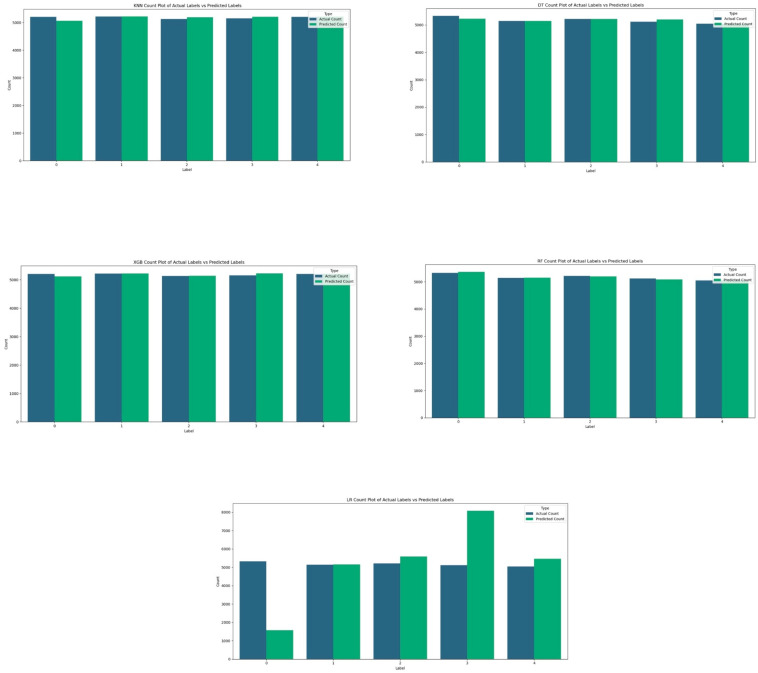
The results in comparing actual and predicted values across the four attack types using XGB, DT, RF, KNN, and LR.

In summary, the evaluation of various classifiers, including Logistic Regression (LR), Random Forest (RF), Decision Tree (DT), K-Nearest Neighbors (KNN), and XGBoost (XGB), reveals notable differences in their performance metrics. LR exhibits moderate accuracy at 82.98% with a relatively longer training time (95.6417 s) and moderate prediction time (0.4987 s). RF and XGB show the highest accuracy at 98.54% and 98.89%, respectively, with RF has a shorter training time (30.86 s) and prediction time (0.0311 s) compared to XGB. DT and KNN demonstrate good accuracy at 97.61% and 98.87%, respectively, with DT having the shortest training time (6.8637 s) among all classifiers. However, KNN exhibits a longer prediction time (0.9469 s). Overall, RF and XGB emerge as top performers in terms of accuracy, with RF being more efficient in terms of training and prediction times. Additionally, we achieved high performance when comparing actual and predicted values across the five classes shown in Figure 8. This outcome indicates that the model can consistently and accurately identify each class, delivering reliable results.

Table 12 summarizes different approaches for detecting IoT botnet activity using machine learning. For example, ref. [13] utilized a Deep Neural Network on the IoT-23 dataset, achieving an accuracy of 93% with a prediction time of 0.2 s. Their approach did not utilize the multiclass classification technique but included cross-validation and real-time capabilities.Ref. [34] employed AdaBoost on the same dataset, achieving a higher accuracy of 99.9% with a much faster prediction time of 0.02156 s, although they did not categorize the attacks nor use real-time capabilities.Ref. [14] used a neural network with multiclass classification on the IoT-23 dataset but did not report the exact accuracy or prediction time. Our work distinguishes itself by utilizing PySpark with random forest (RF) and XGBoost (XGB) for real-time detection, achieving a high accuracy of 98.9% using XGB with a prediction time of 0.0311 s. Our method supports multiclass classification and includes cross-validation, ensuring a robust and responsive solution essential for effective intrusion detection systems (IDSs) in IoT environments. Unlike [13], which has a longer prediction time of 0.2 s, our method ensures a quicker response, essential for the detection of IoT security threats in real time. In reference [34], binary classification may achieve near-perfect accuracy due to the specific data distribution of the problem. However, our approach focuses on detecting various types of attacks in the IoT-23 dataset, resulting in consistently high accuracy across multiple classes. Compared to Alghamdi et al. [14], our method not only reports precise accuracy metrics but also demonstrates superior prediction time, making it highly suitable for real-world IoT applications.

The proposed models have demonstrated strong detection capabilities in real-time against four types of attacks: DDoS, Okiru, Horizontal Port Scan, and unknown attack types. With very high precision, recall, and F1 scores, these models demonstrate robustness in handling diverse attack scenarios within IoT network traffic. This suggests that they can effectively manage the complexities of real-time intrusion detection in IoT environments. The previous analysis confirms that the proposed models are well equipped to handle these IoT attacks, ensuring reliable and efficient network security.

Utilizing PySpark in our methodology not only facilitates the efficient real-time processing but also substantially decreases the prediction time, enabling quick and precise detection of IoT botnet activity. PySpark accomplishes this by utilizing its powerful distributed computing capabilities, making use of the Apache Spark framework to distribute data processing jobs across a cluster of computers. The parallel execution concept enhances computational speed by partitioning the effort and facilitating the concurrent processing of extensive datasets. Furthermore, PySpark employs in-memory processing using RDDs (resilient distributed datasets) and DataFrames. These data structures store data in memory and enhance data retrieval and manipulation. These characteristics reduce the amount of disk input/output operations and optimize the execution of machine learning algorithms, hence improving the speed of predictions. Moreover, PySpark’s MLlib package offers enhanced versions of machine learning algorithms specifically intended for distributed computing, guaranteeing quick training, and prediction of models. PySpark, when combined with its ability to handle IoT data streams in real-time, provides the prompt mitigation of security concerns, hence greatly enhancing the overall security of IoT networks.

## 6. Conclusions

This study introduces a real-time intrusion detection system (IDS) tailored for identifying Internet of Things (IoT) attacks using multiclass classification models within the PySpark framework. By employing five machine learning algorithms and using the OneVsRest (OVR) technique, our approach aims to boost detection accuracy while minimizing the prediction time. Through thorough experimentation on the IoT-23 dataset, comprising network traffic from smart home IoT devices, we assessed the performance of various classifiers. Our findings show that extreme gradient boosting (XGB) attained a high accuracy of 98.89%, demonstrating a high performance in classifying IoT network traffic data. In terms of prediction time, random forest (RF) exhibited the most efficient training and prediction times, with a prediction time of merely 0.0311 s. These outcomes underscore the effectiveness of our proposed method in accurately categorizing network traffic and promptly detecting IoT threats in real-time. Overall, this paper makes a valuable contribution to cybersecurity by presenting a robust IDS framework tailored for IoT environments, leveraging machine learning algorithms and the PySpark architecture. The achieved high accuracy and swift prediction time underscore the potential of our approach for real-time intrusion detection in IoT networks. Future research may explore additional optimization strategies and validate the proposed method on larger and more diverse datasets to ascertain its efficacy in practical scenarios.

## Figures and Tables

**Table 1:** IoT 23 dataset features and counts.

IoT 23 Features	Counts
Horizontal Port Scan Fragment	825,939
Okiru	262,690
Benign	197,809
DDoS	138,777
Attack	3915
C & C Heartbeat Attack	349
C & C File Download	43
C & C Torii	30
File Download	13
C & C Heartbeat with File Download	8
C & C Mirai	1

**Table 2:** LR evaluation metrics.

Class	Precision (%)	Recall (%)	F1 Score (%)
Benign 0	96.28	28.69	44.21
DDoS 1	99.73	99.92	99.83
Okiru 2	89.26	95.80	92.42
PartOfAHorizontalPortScan 3	57.14	90.86	70.16
Unknown attack 4	95.34	100	97.62
Accuracy	82.98		
Cross validation (5-fold) accuracy	80.32		

**Table 3:** LR training and prediction time.

Task	Time (Seconds)
Training Time	95.6417
Prediction Time	0.4987

**Table 4:** RF evaluation metrics.

Class	Precision (%)	Recall (%)	F1 Score (%)
Benign 0	97.79	95.51	96.64
DDoS 1	99.85	100	99.92
Okiru 2	99.72	99.64	99.68
PartOfAHorizontalPortScan 3	95.47	97.56	96.50
Unknown attack 4	99.87	99.98	99.92
Accuracy	98.54		
Cross validation (5-fold) accuracy	98.32		

**Table 5:** RF training and prediction time.

Task	Time (Seconds)
Training time (s)	30.86
Prediction time (s)	0.0311

**Table 6:** DT evaluation metrics.

Class	Precision (%)	Recall (%)	F1 Score (%)
Benign 0	97.40	90.83	94.00
DDoS 1	99.86	100.00	99.93
Okiru 2	99.62	100.00	99.81
PartOfAHorizontalPortScan 3	91.51	97.25	94.29
Unknown attack 4	99.87	99.96	99.91
Accuracy	97.61		
Cross-validation (5-fold) accuracy	96.57		

**Table 7:** DT training and prediction time.

Task	Time (Seconds)
Training Time (s)	6.8637
Prediction Time (s)	0.0804

**Table 8:** KNN evaluation metrics.

Class	Precision (%)	Recall (%)	F1 Score (%)
Benign 0	97.88	96.51	97.19
DDoS 1	99.89	100.00	99.94
Okiru 2	99.65	100.00	99.83
PartOfAHorizontalPortScan 3	97.19	97.84	97.52
Unknown attack 4	99.70	99.96	99.83
Accuracy	98.87		
Cross-validation (5-fold) accuracy	98.55		

**Table 9:** KNN training and prediction time.

Task	Time (Seconds)
Training time (s)	10.9469
Prediction time (s)	0.9469

**Table 10:** XGB evaluation metrics.

Class	Precision (%)	Recall (%)	F1 Score (%)
Benign 0	98.05	96.37	97.20
DDoS 1	99.96	100.00	99.98
Okiru 2	99.96	99.96	99.90
PartOfAHorizontalPortScan 3	96.70	98.12	97.41
Unknown attack 4	99.96	100.00	99.98
Accuracy	98.89		
Cross-validation (5-fold) accuracy	98.61		

**Table 11:** Training and prediction times.

Task	Time (Seconds)
Training time	50.3426
Prediction time	0.7381

**Table 12:** Comparison of our approach to different approaches.

Reference	Approach	Dataset	Multiclass Classification	Cross-Validation	Real-Time	Prediction Time	Evaluation of Metrics	Highest Accuracy
[13]	Deep Neural Network	IoT-23	Not used	*√*	*√*	0.2 s	*√*	93%
[34]	AdaBoost	IoT-23	Not used	*√*	-	0.02156 s	*√*	99.9%
[14]	Neural network	IoT-23	Yes	*√*	*√*	-	*√*	Exact accuracy not reported
Our work	PySpark (RF, XGB)	IoT-23	Yes	*√*	*√*	0.0311 s (using RF)	*√*	98.9% (using XGB)

## Data Availability

Data are available upon request.

## References

[1] Shen G., Liu B. (2011). The visions, technologies, applications and security issues of Internet of Things. Proceedings of the 2011 International Conference on E-Business and E-Government (ICEE).

[2] Vailshery L.S. Global IoT and Non-IoT Connections 2010–2025. Statista. https://www.statista.com/statistics/1101442/iot-number-of-connected-devices-worldwide/.

[3] Cisco Newsroom Powering an Inclusive, Digital Future for All. Newsroom. January 2023. https://newsroom.cisco.com/c/r/newsroom/en/us/a/y2023/m01/powering-an-inclusive-digital-future-for-all.html.

[4] Gupta S., Vanjale S. (2020). Cyber Security Measures for Internet of Things Devices. Int. J. Eng. Res. Technol..

[5] Hung M. (2017). Leading the IoT: Gartner Insights on How to Lead in a Connected World. https://www.gartner.com/imagesrv/books/iot/iotEbook_digital.pdf.

[6] Rahman S.A., Tout H., Talhi C., Mourad A. (2020). Internet of Things Intrusion Detection: Centralized, On-Device, or Federated Learning?. IEEE Netw..

[7] Zhong M., Zhou Y., Chen G. (2021). Sequential Model Based Intrusion Detection System for IoT Servers Using Deep Learning Methods. Sensors.

[8] Chatterjee A., Ahmed B.S. (2022). IoT anomaly detection methods and applications: A survey. Internet Things.

[9] Abbas A., Khan M.A., Latif S.e.a. (2022). A New Ensemble-Based Intrusion Detection System for Internet of Things. Arab. J. Sci. Eng..

[10] Liu H., Han D., Cui M., Li K.C., Souri A., Shojafar M. (2022). IdenMultiSig: Identity-Based Decentralized Multi-Signature in Internet of Things. IEEE Trans. Comput. Soc. Syst..

[11] Jeelani F., Rai D.S., Maithani A., Gupta S. The Detection of IoT Botnet using Machine Learning on IoT-23 Dataset. Proceedings of the 2022 2nd International Conference on Innovative Practices in Technology and Management (ICIPTM).

[12] Nanthiya D., Keerthika P., Gopal S.B., Kayalvizhi S.B., Raja T., Priya R.S. SVM Based DDoS Attack Detection in IoT Using Iot-23 Botnet Dataset. Proceedings of the 2021 Innovations in Power and Advanced Computing Technologies (i-PACT).

[13] Bhandari G., Lyth A., Shalaginov A., Grønli T.M. (2023). Distributed Deep Neural-Network-Based Middleware for Cyber-Attacks Detection in Smart IoT Ecosystem: A Novel Framework and Performance Evaluation Approach. Electronics.

[14] Alghamdi R., Bellaiche M. A Deep Intrusion Detection System in Lambda Architecture Based on Edge Cloud Computing for IoT. Proceedings of the 2021 4th International Conference on Artificial Intelligence and Big Data (ICAIBD).

[15] Patil N.V., Rama Krishna C., Kumar K. (2020). S-DDoS: Apache spark based real-time DDoS detection system. J. Intell. Fuzzy Syst..

[16] Othman D.M.S., Hicham R., Zoulikha M.M. (2020). An efficient spark-based network anomaly detection. Int. J. Comput. Digit. Syst..

[17] Yang L., Cai M., Duan Y., Yang X. Intrusion detection based on approximate information entropy for random forest classification. Proceedings of the 2019 4th International Conference on Big Data and Computing (ICBDC 2019).

[18] Anthi E., Williams L., Słowińska M., Theodorakopoulos G., Burnap P. (2020). A Supervised Intrusion Detection System for Smart Home IoT Devices. https://orca.cardiff.ac.uk/id/eprint/123767/1/A%20Supervised%20Intrusion%20Detection%20System%20for%20SmartHome%20IoT%20Devices.pdf.

[19] Ahmad R., Alsmadi I. (2021). Machine learning approaches to IoT security: A systematic literature review. Internet Things.

[20] Pwint P.H., Shwe T. Network Traffic Anomaly Detection based on Apache Spark. Proceedings of the 2019 International Conference on Advanced Information Technologies (ICAIT).

[21] Zaharia M., Xin R., Wendell P., Das T., Armbrust M., Dave A., Meng X., Rosen J., Venkataraman S., Franklin M.J. (2016). Apache Spark. ACM Comput. Surv..

[22] Apache Spark Home Page. http://spark.apache.org/.

[23] Choudhary P., Garg K. Comparative analysis of Spark and Hadoop through imputation of data on big datasets. Proceedings of the 2021 IEEE Bombay Section Signature Conference (IBSSC).

[24] Kumar K., Sharma N., Ali A. Machine Learning Solutions for Investigating Streams Data using Distributed Frameworks: Literature Review. Proceedings of the 2021 IEEE Asia-Pacific Conference on Computer Science and Data Engineering (CSDE).

[25] Tun M., Nyaung D., Phyu M. Performance evaluation of intrusion detection streaming transactions using Apache Kafka and Spark Streaming. Proceedings of the Proceedings of the 2019 International Conference on Advanced Information Technologies (ICAIT).

[26] Karau H., Konwinski A., Wendell P., Zaharia M. (2015). Learning Spark: Lightning-Fast Big Data Analysis.

[27] Meng X., Bradley J., Yavuz B., Sparks E., Venkataraman S., Liu D., Freeman J., Tsai D., Amde M., Owen S. (2016). MLlib: Machine Learning in Apache Spark. J. Mach. Learn. Res..

[28] IoT-23 Dataset. https://www.stratosphereips.org/datasets-iot23.

[29] Manzano R., Zaman M., Goel N., Naik K., Joshi R. (2022). Towards Developing a Robust Intrusion Detection Model Using Hadoop–Spark and Data Augmentation for IoT Networks. Sensors.

[30] Mirza A.H. Computer Network Intrusion Detection using Various Classifiers and Ensemble Learning. Proceedings of the 2018 26th Signal Processing and Communications Applications Conference (SIU).

[31] Apache Spark Classification and Regression. https://spark.apache.org/docs/latest/ml-classification-regression.html.

[32] XGBoost XGBoost JVM Package. https://xgboost.readthedocs.io/en/stable/jvm/index.html.

[33] Cunningham P., Delany S.J. (2006). k-Nearest Neighbour Classifiers. ACM Comput. Surv. (CSUR).

[34] Hazman C., Guezzaz A., Benkirane S., Azrour M. (2022). lIDS-SIoEL: Intrusion detection framework for IoT-based smart environments security using ensemble learning. Clust. Comput..

